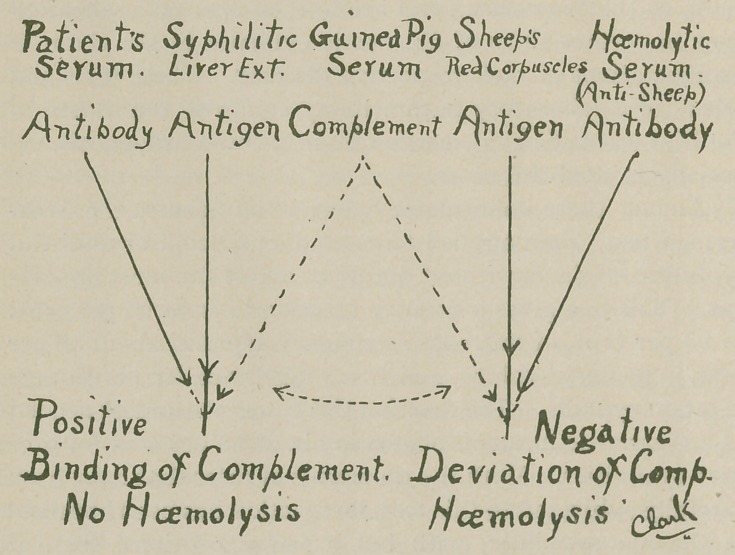# Syphilis, with Special Reference to Recent Experimental Work

**Published:** 1909-11-15

**Authors:** Henry Rockwell Varney

**Affiliations:** Detroit


### SYPHILIS, WITH SPECIAL REFERENCE TO RECENT EXPERIMENTAL WORK.

BY HENRY ROCKWELL VARNEY, M.D., DETROIT.

Clinical Professor and Lecturer on Dermatology and Syphilology in the Detroit College of Medicine; Dermatologist to Harper Hospital; State Reform School; Dearborn Retreat. Member of the International Congress of Dermatologists.

Read before the Michigan First District Dental Society Oct. 14th, 1909.

Much has been written upon Syphilis, much more should be written, and much must be written before we can become united in the successful management and control of this disease.

A consideration of some of the following facts, abstracted from recent research, should be of interest in every special branch of medicine because every Physician and Dentist must consider this disease in his given field of work. It is by such considerations that we are to have a more definite understanding of this disease and thereby prevent not only its spread but some of the final results of its ravages, which may be permanent disfigurements, to the patients deformity or they may become helpless, permanent inmates of State Institutions.

The fruits of recent research have been so productive in adding to our knowledge of syphilis, that our text books on the subject, even with their frequent new additions, have been unable to keep abreast of the times.

The true etiological factor, the spirochaeta pallida of syphilis is now generally accepted; finding them in questionable lesions, is of inestimable assistance to a correct diagnosis. Animal experimentation has also added volumes of accurate knowledge pertaining to all phases of the disease. It is now demonstrated that the same organism is capable of producing the same disease in the higher order of apes as is produced in the human. Without doubt the most valuable knowledge obtained through animal experimentation is that pertaining to treatment, both prophylactic and constitutional. We no longer treat syphilis symptomatically, with no knowledge of its cause more than it was infectious; we no longer instruct our students to wait until the secondary symptoms of syphilis, symptoms of general infection, present themselves, before a diagnosis is made,and specific treatment of questionable sores is begun. We now know that the single or multiple chancreoid, so diagnosed clinically often are a mixed infection and being so diagnosed clinically is often discarded by the physician. Because of the attitude of the physician, mild secondary symptoms escape the notice of the patient who, later in life, presents grave manifestations of the disease. We now have diagnostic means, not only in finding the organism in questionable lesions, but in demonstrating protective substances in the blood serum in which infection has taken place.

In taking up the recent advancements in the knowledge of this disease let us first consider the organism. The etiology of syphilis for centuries has been the deepest of mysteries and more than a hundred causes have been heralded but none have stood the test of Kochs Law. The difficulties in recognizing and isolating the organism have been many, yet the disease is produced in animals, and the organism demonstrated in the infected animal during all stages of the disease, and in all lesions of it. This makes clear that the spirochaeta pallida, which is now being classified as a flagellate-like protozoa, is the etiological factor of syphilis.

The life of the organism is but a few hours, after leaving the body, demonstrating the slight risk from infection to which we are all often subjected, from the use of articles in common with the syphilitics.

The virulence of syphilitic virus can be preserved for some time in glycerine, 35 days (Jancko's experiments) as small pox vaccine, yet the organisms die rapidly in glycerine.

This virus or the virus passed through a Chamberlain filter is capable of producing infection in monkeys while the organism is unable to pass through the filter.

The greatest difficulty in demonstrating the organism arises from the fact that there are a number of common spirochaetes that closely resemble the spirochaeta pallida. Three of the most troublesome spirochaeta from point of differentiation are found in the buccal cavity.

Two of the most common, the spirochaeta refringens and spirochaeta buccalis can be differentiated and excluded from the spirochaeta pallida by staining with Giemsa stain. The pallida takes a pale red while the refringen and buccalis stain blue.

The spirochaeta dentium and the pseudo pallida both take the red stain and must be differentiated by their character of growth. The spirochaeta dentium can be grown on serum agar, is slightly thicker than the pallida and its curves more angular (saw like), while the spirochaeta pallida has a more corkscrew curve.

The spirochaeta pseudo gives far the most trouble and many workers maintain that it is impossible to differentiate it from the pallida if the examination is confined to smears obtained only from the mouth fluids.

If serum to be examined can be obtained from lesions other than the mouth, one may save time by detecting the living spirochaeta by means of the Darkfield Illumination.

They are seen by this means of illuminating as very thin and regular corkscrew-like spirals upon a dark background. They have a slow forward and backward motion and a very active rotation in longitudinal axis, bending motion of the whole body or only on end without changing their position in the field. They can be kept alive for days, while the virus loses its virulence in 6 to 10 hours.

The second important recent discovery of syphilis has been animal experimentation. It is proven that not only the highest forms of apes can be inoculated with the organisms of the disease, but that the lower forms of apes are susceptible. Great honor is due to Professor Neisser, for his extensive experimentations upon apes in this disease. He spent seven years in Java, the sole purpose of which was to study animal experimentation of this disease with the animal in its natural habitation. The important results of his work are that he proved that the animals, just as the human race are susceptible to the virus of syphilis. Yet no animal has been found that is as susceptible as man. A primary sore develops, after which the whole body shows signs of general infection, characterized by cutaneous eruptions, general adenitis and involvement of the mucous membrane, especially the buccal cavity. Marked difference in the development of constitutional symptoms was noted in different species of apes; the lower forms seldom showed secondary symptoms and when shown they were very difficult to inoculate; only upon certain parts of the body, such as the eye-brows, genitals, and scrotum, could they be inoculated, while the higher species could be inoculated on any or all parts of the body. During these experiments, much attention was given to the development, course, and severity of the disease from different strains of the virus, obtained from different lesions of different stages of the disease, both human and animal.

No difference was noted in any stage of the disease, as influenced by the manner in which the animal was inoculated, except that the inoculations were more successful when the virus did not enter the blood stream. The disease ran the same course, with the same virulence, regardless of the stage of the disease, or from the lesion from which the virus was taken, whether primary or tertiary, demonstrating that the organism does not lose its virulence or become attenuated with age, but is capable of producing a typical infection, running the same course, when the organism is implanted upon a new field. The course of the disease then, if influenced at all, is brought about by the manner of implanting the virus, rather than by the strain. It also shows that any and all syphilitic manifestations, of whatever stage of the disease, harbor the organism and are directly produced by it. The area of inoculation or the initial sore of animals is not characteristic with its induration, as is the case in man, and oftentimes, the inoculation has been considered unsuccessful, so slight were the manifestations, until the animal was killed, and the organism found in the internal organs.

Inoculations of infected blood, with but few exceptions, have been unsuccessful, while with the seme , Finger has twice produced the disease. Further experimentation along this line may be productive of definite knowledge in regard to the transmission of the disease by parental infection.

Infected animals have also given us much knowledge in regard to the destruction of the organism by physical and chemical means. These prophylactic precautions, obtained by drugs, will soon become generally known as they are in France, and will without doubt, be of unquestionable value as a prophylactic measure in arresting the onslaught of this disease. The advice governing the dispensing and use of such means should be most guarded, and come from the physician.

Metchnikoff, by both man and animal experimentation, has shown that by the application of a calomel ointment applied at the site of inoculation, even after a period of from one to three hours, further syphilitic infection can be aborted by the destruction of the organism. He, Neisser and others are advocating the use of such medical means as a general prophylactic precaution against the spread of this disease, not in the spirit of encouraging evil, or in any way removing the fear of contracting the disease, which has been a most powerful safeguard to those who have been taught its dangers but they advocate it in the spirit of true physicians whose highest aims are to protect all humanity from any disease, foreseeing that, by such prophylactic measures, the coming generations of all nations will be spared much sorrow and suffering.

From these scientific facts we can gain much in simple means in the prevention of infection. Dentists who are working in infected mouths every day cannot protect their fingers as can the physician with rubber gloves. They therefore must depend upon antiseptic precautions in the form of solutions before and after operation. A prophylactic precaution which is demonstrated scientifically worth while, is the application of Calomel ointment well rubbed in before beginning work in the suspected mouth. This will without question furnish the best protection to the cracked condition of the skin around the finger nail, the most common location of infected and extergenital chancre.

Results in systemic treatment of the disease with our present knowledge, are still most uncertain, yet far more certain than even five years ago. The patient is treated early with drugs that in experiments with animals, have controlled and cured the disease. The symptoms all disappear, the patient goes on through the average length of life with no further manifestations of the disease. Then again we may give the same drugs, at the same stage of the disease, the same duration of time, discharge the patient, clinically cured, and in ten or twenty years later, a parasyphilitic condition develops. The cure of syphilis then, does not depend upon any one drug, or the salts of any number of drugs, given any length of time, or administered in any one or all of the approved ways, but upon nature's means of resisting this disease, on the part of each individual patient, in the development of antibodies, nature's protective substances are Nature's means of preventing the organism and its toxins from attacking vital cell structures. Just what are our effects upon nature's efforts in the means we are employing especially in administering drugs, is a most important problem in the successful treatment of this disease.

Animals infected with the disease can be treated, symptomatically cured then killed, or other animals may be inoculated, from the infected animal, or they can be re-inoculated, and by such procedure ascertain what has been the effect of different treatments in the cure of the disease. Mercury still holds first place in its action on the organism and its virus arsenic second, iodide of Potassium third. Animals that have been infected and re-inoculated, after treatments have been given, are considered cured, or syphilis free, and further, in second inoculation, the sore, the seat of inoculation, is all that is manifest of the disease. No secondary symptoms are presented, which demonstrates a partial immunization, brought about by the first infection, and the immunization continues after all active infection of the disease disappears, as in the vaccination for smallpox.

Further research in the study of the blood changes of the syphilitic, as in the Wasserman test, may make this important phase of the disease clear as in an accurate demonstration of the antigens, in other words, when we can determine for our syphilitic that he is syphilis free. This brings us to the third and more recent advancement in this disease.

The recent demonstration of substance in blood sera from certain diseases leads us to accept the presence of such substances as positive indicators of the disease due to a specific cause. Positive reactions to the Widal test, Tuberculin, or the Wasserman test are in their specific application very important links of diagnostic evidence in the chain of manifestations of their respective diseases. These tests and others of a laboratory nature today comprise the source of the best scientific evidence to obtain a correct diagnosis, especially in doubtful cases.

Among these confirmatory diagnostic agents, the Wasserman test, when applied to cases of suspected or doubtful syphilitic origin, is without question, one of the most important. This test gives a positive reaction in from 90 per cent. to 95 per cent. of untreated secondary syphilis, about 50 per cent. in tertiary syphilis, and a very much smaller percentage in latent syphilis. The reaction therefore, when most needed as a diagnostic means is shown in about 50 per cent. of positive syphilitics. With the thousands of cases that have been carefully subjected to this test, no reaction has been obtained in the non-syphilitic, with but a few exceptions, such as sleeping-sickness, Yaws, Leprosy and Scarlet fever. These exceptions, do not decrease the value of the test, because of the ability to easily exclude clinically the diseases mentioned.

The time has come when we, as physicians and dentists must become familiar with the substances in the blood, that enter into different reactions which are being commonly employed in this and other reactions, for they are essential in the great field of modern medicine dealing with the process of immunization.

The Wasserman test which we will briefly outline, depends upon two systems, a bacteriolytic and a hemolytic. These two systems are necessary to measure and observe the phenomenon-fixation or deviation of complement. The (a) or bacteriolytic system consists of 3 factors namely—1st antibody (patient's serum) 2nd antigen (syph. ext.) 3rd complement (G. pig serum.) The (b) Hemolytic system in turn consists of 3 factors namely: (5) antibody (hemolytic anti-sheep rabbit serum), (4) antigen (sheep's corpuscles) and (3) complement (G. pig serum) which is common to both. (See chart.)

1st—the patient's blood is drawn, the serum separated and heated at 56 degrees C. ½ hour the 2nd factor or antigen is an alcoholic extract of livér from stillborn syphilitic infant: 3rd or complement is the fresh clear serum from a normal guinea pig and is common to both systems. 4th Hemolytic antigen or sheep's blood corpuscles are obtained by drawing a quantity of sheep's blood into 1 per cent. sodium citrate in salt solution, centrifuging, decanting, taking up in salt solution, centrifuging, and again taking up in salt solution and centrifuging. The thoroughly washed corpuscles are then diluted in salt solution, 1:100. 5th factor, or Hemolytic antibody is secured from blood of rabbit which has been immunized to sheep's red blood corpuscles by 5 injections at intervals of four to six days, and then inactivated by heating at 56.0. C. ½ hour.

The factors of the Hemolytic system are first standardized by determining the amount of complement (3) necessary to reactivate one unit of antibody (5) sufficiently to liberate the Hemoglobin from a unit of antigen (4) in ten minutes at room temperature.

The antigen in the Bacteriolytic system is in turn standardized to the units used in the Hemolytic system by using sera from a known positive syphilitic and a known normal.

Having now the material for making the test, we are ready to put the five reagents together. One drop of the suspected serum of the patient, 1 unit of complement and 1 unit of antigen are placed in one tube. Another tube containing 1 drop of positive syphilitic serum, and also one containing one drop of a known non-syphilitic serum with units of the other two reagents in each are placed together. By so doing, the patient's serum is controlled by a positive, and a negative control serum. No reaction that is not so controlled can be trusted for accuracy. These sets are placed in an incubator for 20 minutes, after which time, 1 unit of each of sheep's corpuscles and Hemolytic serum are added to all of the tubes which are then allowed to stand at room temperature 10 minutes. If an antibody be present, the complement will be fixed, so that when the red blood corpuscles and the hemolytic serum are added, there is no complement to unite with the corpuscles and the serum, therefore, no hemolysis takes place. If no antibody is present the complement is not fixed, and is therefore free to react with the hemolytic serum of the rabbit and the corpuscles of the sheep,— and hemolysis takes place, a check-off on the test being observed in the positive and negative controls.

After considering the substance which enter the Wasserman reaction, the manner in which they are obtained, the standardization of each and the constant deterioration of many of these reagents one is readily convinced that the test is most difficult. It requires a skilled technician, who is versed in blood work, and a well equipped laboratory. Months are required to become skilled in each step of the test. The guinea pig serum containing the complement must be fresh, which means every twenty-four hours, the hemolytic serum obtained from the immunized rabbit can be depended upon for an indefinite period only if kept in an ice chest. The red blood corpuscles of the sheep begin to disintegrate and are useless in three days. It is therefore a most extraordinarily complicated procedure—and expensive, due to the, as yet unavoidably unstable ingredients.

The original Wasserman reaction, with a number of recommended modifications while laborious and complicated, is reliable as a general diagnostic measure, but unfortunately its application is at present limited to the larger medical centres.

Many investigators have sought to simplify Wasserman's method, so that it can be generally applied, and while we have as yet none that will stand the test for accuracy, we have reason to believe that practical simplifications will be perfected in the near future.

The serum of the syphilitic contains, according to experiments of many investigators, an excess of globulins as compared with normal sera, and those of most other diseases. This fact has led many to attempt to precipitate these globulins by various chemical means so as to be of diagnostic value, either in quality or quantity of the precipitants formed. Those who have exhaustively compared the precipitation reaction, with the fixation of complement reaction are of the opinion that while one may become skilled through the extensive use of the precipitation test it is impracticable for general use in the hands of the physician who seldom needs to employ it. The precipitation reaction while simple is very delicate, and can not be considered as reliable in the doubtful case where an accurate test is most needed.

The author has tested more than 400 bloods with several reagents recommended by various workers in the precipitation test, and with a soluble salt previously reported by him. This reagent Taurin proved to be the most accurate. It is white, crystalline, soluble bile salt and when used in different dilutions precipitates the globulins in larger quantities, in shorter time and results in a precipitate of a different character, than any other substance as yet employed. The sera examined by this test were numbered with no knowledge of whether the blood was or was not syphilitic and the reaction verified by the history of infection, or most often by actual objective clinical symptoms of syphilis.

The author's experience with Taurin leads him to think that it is a simple means of strengthening the diagnostic evidence in doubtful, clinically manifested cases. In summing up the reports of 150 skilled laboratory workers on the Taurin reaction, the evidence isthat the casual worker's interpretation of the precipitate is not accurate or definite enough to be relied upon.

The precipitation reaction in syphilitic blood, has been demonstrated by Noguchi, Neubauer, Solman, Porges, Elias, and others. Noguchi precipated the globulins in the spinal fluid of the syphilitic with butyric acid. If the serum so tested, is normal, there is slight, if any, opalescence; but if syphilitic, a prompt cloudiness appears, and it soon becomes flocculent. The results reported by Noguchi have compared well in accuracy with those of the original Wassermann, and his reports have been comfirmed by Fox, Dr. Santos-Saxe, and others. This test, while far more simple than the Wassermann presents some objections, viz: that of obtaining the serum from the spinal canal, and also the very disagreeable odor of butyric making it impracticable for office work.

Still another type of reaction depends upon the increase in globulin content is a color test reported by Schurman. We were unable to confirm his findings, but feel that such a test has promising possibilities. The author has done some preliminary work with several color reagents singly and in combinations, but as yet without success.

At present, the serum diagnosis of syphilis cannot be considered perfected, yet much disputed diagnostic evidence is now considered reliable. Its assistance is of inestimable value in differential diagnosis of the obscure, latent syphilis, especially of the'nervous system, where no history of positive clinical symptoms of the infection is known.

Early diagnosis means weeks of early treatment and with vigorous early treatment the duration of the disease is materially shortened and the percentage of cures is accordingly larger. If the Wassermann reaction can be obtained at an early period of general, systemic infection,coupled with the finding of the specific organism, we have not only made great advancement in the early diagnosis of this disease but also in its control. This evidence can be considered only clinical and yet without definite proof. The most important value of the Wasserman reaction may not be that of diagnosis. If we can prove after continued negative reactions of a patient's serum, that the patient who has been afflicted with the disease is entirely free, the test will become of still greater value to patient and physician.

Only by carefully testing many sera for a long period of time can this be accomplished. Many investigators are now maintaining that a patient's serum giving a positive Wassermann reaction may become negative, after vigorous treatment with mercury. They also maintain that a positive reaction means positive syphilis and further treatment.

Much time must be spent on further study of syphilitic sera before the governing of our treatment by reaction can be accepted as absolute. Just how much power of resistance the average normal individual possesses against this infection at a certain age, and just what the effect of our specific treatment is upon the infection, or upon nature's means of combating the infection, is yet a vast unknown field. Why some patients run such an apparently mild course, while others are most severe and destructive under the same conditions and treatment, can only be known when we are able to determine the value and cause of individual resistance to this specific infection.

If again we can be guided by the reaction, in our management and treatment of this disease, society and the coming generations will be inestimably benefitted. We can then advise our patient not only regarding the safety of marriage but will be able to state whether he can have healthy children, if we have a definite practical means of knowing when he is cured. The only absolute proof we now possess of the surety of our patients being syphilic free, is that he may become re-infected. If all patients that show the reaction still have active syphilis, we are curing only a small percentage of our syhpilitics. Yet on the other hand, if our patient can be reinfected, we can consider syphilis curable.

In the latent stages of syphilis, the patient shows some proof of immunity. Lesser estimated the presence or absence of the reaction, tabulating them by years, since the patient became infected. The reaction was present in about this ratio:

The first two years, it was found in over 70 per cent.: from the third to thirteenth year about 50 per cent.; from the thirteenth to thirty-fifth year 11 per cent. and only rarely after the thirty-fifth year of infection. He also reports that in 86 per cent. of those giving a positive reaction, marked decrease in the number and extent of the positive reactions is so rendered by treatment. He maintains that the longer the infection has existed the more likely are relapses, and the more constant is the reaction positive. The earlier the treatment, the longer continued, the larger is the percentage of negative reaction that continue negative.

Citron concludes from work on a large series of cases as follows: First-the constant finding of the reaction indicates positive action syphilis; second-that poorly treated or untreated syphilitics show the reaction after many years; third-if the reaction is present, there is active syphilitic infection, and treatment should be continued.

Blaschko has also reported much in the systematic examination of the serum, before, and during various methods of treatment in the different stages of the disease. He believes, and maintains, that the treatment should be continued as long as a positive reaction is obtained, and that repeated tests of the serum after it has become negative is necessary. If the reaction reappears treatment should be given even in the absence of all other symptoms of the disease.

CONCLUSION.

While for centuries, many hundreds of causes of syphilis have been heralded, none have thus far stood the test of Koch's Law.

The Spirochaeta Pallida, a flagellate-like Protozoa is found in all lesions and stages of the disease. The organism is capable of producing the disease in animals and is recovered in organs of the infect-animal.

The isolation and the growth of the organism in pure culture has not as yet been accomplished.

The difficulty in isolation and growth of the organism makes it most difficult to differentiate the spirochaeta pallida from many other common spirochaeta, especially those found in the oral cavity.

Examination of serum from suspicious lesions by the Darkfield illumination affords an easy and quick means of detecting the spirochaeta pallida in its active state.

The shape and motion can readily be detected by this means.

The organism can be kept alive for days in abundant collected material for media, at body temperature, but dies rapidly after leaving the body.

Animal experimentations demonstrate that all lesions harbor the organism, and are capable of producing the diseases by inoculations. Inoculations are more successful when the virus does not enter the blood stream.

The efficiency of drugs in the control and cure of this disease are now scientifically known. Mercury hold first place in the prevention, control, and cure of Syphilis, Arsenic second, and Iodide of Potassium third.

Prophylactic precautions for the physician and dentist are now scientifically made definite.

Demonstration of the presence or absence of protective substances in the blood of the syphilitic, as in the Wassermann reaction, is of inestimable value in diagnosis and treatment.

The Wassermann reaction shows a positive reaction in from 90 per cent. to 95 per cent. of untreated secondary syphilis.

A negative Wassermann does not always mean the absence of syphilis as a small percentage of syphilitics fail to show the reaction.

Two or more Wassermann tests should be run in all doubtful cases. Every reaction being carefully controlled by a known positive syphilitic blood and a known non-syphilitic blood. No test can be depended upon for accuracy that is not so controlled.

Simplifications of the original Wassermann test is sure to be forthcoming, and time is only necessary, to prove the accuracy of many now advocated. Further research in the testing of the blood of the syphilitic, before and during treatment in all stages of the disease, will without doubt, give us definite means, whereby we can tell, when our syphilitic is syphilis free.

To L. T. Clarke, B.S., I wish to express my thanks for not only the chart, but for valuable laboratory assistance in presenting this paper.

DISCUSSION.

Dr. C. H. Oakman.—One thing in the paper struck me as very significant and interesting was that the Spirochaeta may be retained in the filter and the virus which passes through the filter has the power of producing syphilis by inoculation. If the spirochaeta is the specific organism of this specific disease, it seems to me as though it would be necessary to introduce that organism, whether subcutaneously or otherwise, in order to produce the disease. If the filtered specific organic extract when applied to the conjunctive might give a reaction similar to the tuberculin test, that seemed to me the strongest point of the paper and I shall be glad if Dr. Varney will speak of this in closing his paper.

As dentists, I think Dr. Varney has given us a very good preparation for us to use daily, providing we operate in mouths that are syphilitic or suspicious, and that is the Calomel Ointment on the hands. We all have hang nails, and more or less abraded hands in spite of all we can do, and I think it should be well for each of us to have a bottle of this ointment.

It was my understanding some years ago that a syphilitic who would contract syphilis for the second time or a person who had had syphilis and was supposed to have been cured, that in a few years later he would develop again the primary sore. I was taught to believe that but very few cases were ever absolutely cured where inoculation has occurred, but as Dr. Varney states here to-night, if you can by the Wassermann test, by making it several times, be satisfied that the patient is syphilitic free, it would stand to reason that if he is absolutely free of syphilis that he must be inoculated again to have it.

In Researches of Rockefeller Institute, New York, there is a very interesting article printed in the Journal of Experimental Medicine, in the March issue of this year which is well worth reading. From what I can find about the Wassermann reaction, it seems to be a most difficult reaction to obtain, as the essayist has already said. I understand it takes some two hours to make the test after the solutions are all at hand, but the technique is so exacting that it takes an expert laboratory man and bacteriologist to perform.

Syphilis, from the dentist's standpoint, is somewhat perplexing to most of us. I once had a dentist say to me, while talking on the subject, "It's funny, none of my patients ever seem to have syphilis, and I have been in practice now eight years, and have never seen a case." His patients were made of different clay from most of ours. He had never seen a case of syphilis in the mouth? The fact is, he had seen many syphilitics but never recognized them. We know syphilis is a disease of civilization, it is often spoken of as a disease of civilization. We know what ravages the disease has caused among the Indians and Negros, where its devastations for ages has been tremendous.

I think Dr. Varney's paper and papers along this line would be a sermon to young men generally, and I also think the clergy and all laymen would be benefitted by hearing such a paper on the subject. We have begun to think that it is almost a crime to go to communion, where perhaps four or five hundred people drink from the same cup. The common drinking cups on trains and at watering points in public buildings are a menace to the public health and should be speedily abolished.

Dr. N. S. Hoff.—I know so very little about the etiology or therapeutics of syphilis that I shall throw no light upon this subject from its scientific or clinical aspect.

As the editor of a dental journal, I always take the liberty of editing the remarks of persons who discuss papers, by eliminating the preliminary remarks which are usually used in commending the essayist of the paper; but I am going to take the liberty to-night of doing the very thing that as an editor I do not allow other people to do, and that is to compliment the essayist, and I do it because I think it is fully justified in this case. I want to say that while I disclaim any particular knowledge of this disease, in a general way, I have followed everything that has been written from a dental standpoint upon this subject, but I have never seen this subject presented in a way that has so impressed me as in this paper that has been read to us to-night. It is a scientific presentation, and for that reason it may not appeal to us as practical, but I believe that if you have carefully listened to this paper, and if, when it is printed you will read, and study it, as Dr. Oakman says he has, you will find in it the basis for definite knowledge concerning this disease, a knowledge which will enable you to value every article that you ever see written upon this subject. After I received this paper I read it through, and I became so much interested in the subject that I located in our journal literature several articles that I remembered having seen on the subject. These articles when I read them in the first place seemed to me quite uninteresting as they gave only the classic symptoms of syphilis and the manifestations that we ought to look for in the mouth, in fact a formal statement of the etiology of the disease in general terms from a clinical standpoint. I did not understand them and they did not appeal to me. But as I re-read them in the light of this paper and this presentation they took on an entirely new meaning to me. I believe Dr. Varney has read before this society to-night the best paper that has ever been presented on this subject before any dental society. It is a paper which will have a permanent educational value for us such as few other papers upon this subject have ever had that have come to my notice. I presume our medical men may be entirely familiar with these facts, but this paper places the whole subject on a basis where any intelligent man, whether phy. sician, dentist or layman can comprehend the subject. We shall also be able to intelligently comprehend what we read upon this subject from other writers who write wholly from the clinical standpoint. A vote of thanks is a very tame expression of my own feelings to Dr. Varney for presenting this subject in this way to us. I shall treasure it as one of the classics in my memory of articles to which I shall always refer for the beginning of any study which I may wish to make on this subject.

I want to say a few things from the dentists' standpoint about this subject. I cannot of course discuss this presentation of Dr. Varney's, because I am neither a scientist nor a physician. Dr. Oakman has already said that this paper practically means that the dentist in practice is to introduce into his practice the diagnosis of this disease as it may come in his hands. We have neither the laboratory nor the skill, as Dr. Varney has said, to do this work. We cannot be expected to do it. In the first place, we should have great difficulty in talking of anything of this sort to our patients; they would resent such interference as impertinent. They will say: What right have you to be suspecting me, or what right have you to be doing such a thing as raising questions as to our conditions or circumstances, even though indications warrant them. We all know how sensitive people are about this disease. If they understood the subject scientifically as Dr. Varney does, and if we all understood it from that standpoint, we should have no difficulty in discussing it freely with our patients, and in so doing enlist their co-operation in preventing its being propagated.

We could not only discuss it with our patients, but even go to the extent of making researches to determine whether a patient was afflicted or not. As we have neither the skill nor appliances, and our patients are not educated to the point of permitting us to do things of this sort, it leaves us only one resort, and that is to fall back upon the specialist, the man who is intelligently doing this work. I am glad we have got such a man as Dr. Varney here in Detroit, and I trust that yo[illegible] men, whenever you have occasion to question a patient's condition on this subject, that you will do what you can to influence him in the speedy determination of so important a matter as this, not only for your own sake but for the benefit of the public and the patient himself. It is one of the most interesting and important subjects that we can take up at this time. It is equally important with that of tuberculosis, which just now seems to be occupying the attention not only of the medical profession but of the laity as well.

The treatment of this disease, while it may be interesting to us as dentists, we have no right professionally to undertake, but that is no reason why we may not understand the therapeutics of this disease. There is no reason why we should not be intelligent about it. It certainly would be unprofessional and discourteous for us to presume, because we knew that our patients had this disease, to prescribe treatment for it. It should be our business, and our pleasure, to refer patients of this sort to a man who is competent to treat it and understands it, and in whom we have the utmost confidence, to take care of it. This being the case, if we as dentists are not capable of either diagnosing this disease or treating it ourselves intelligently and satisfactorily, it becomes imperative when we are suspicious even, of disease of this kind, in any of our patients, to call their attention in some frank, free way to te fact that they should consult a competent person as to their systemic condition. We can do this without exciting nay suspicion that we are trying to overstep the bounds of propriety of our own work. We can do this in a proper way, and it seems to me that it ought to be not only our pleasure but our duty to do this thing for the benefit not only of our patients but humanity in general; and since it is a fact that, as Dr. Oakman has said, we as dentists are not sufficiently intelligent as to this disease, even of the manifestations of it in the mouths of our patients to recognize it by symptoms, which may be not very definite, or may be obscured and of such a character that we cannot differentiate them from other diseases. While we are in this condition as to our knowledge of symptoms of this disease, it ought to be at the same time one reason why we should be more careful as to how we handle all of our patients. We should use at least, renewed precautions in all suspect cases. We are told that there are certain conditions in the secondary stage of syphilis where persons are most dangerous, when the infection is most likely to be transmitted by contact of instruments in various ways, from one patient to another, and I have been always on the lookout for these cases. Of course in the tertiary stage where there are pronounced manifestations that no man can overlook, there is no danger of any of us neglecting proper precautions, but many of the primary lesions are obscured in such a way that we perhaps do not notice them or overlook them. In dealing with conditions of this sort only occasionally, not being wide awake to their manifestations, it is not much wonder that we do sometimes overlook them. I do not see how dentists can be expected to be able to diagnose these conditions with anything like accuracy when many times they are so obscure that even the practiced physician, the best of physicians, cannot determine definitely that it is a syphilitic lesion unless there is a characteristic destruction of the tissues.

Now this brings me to another thought that I want to bring to Dr. Varney's attention, as he, perhaps, being a leader in this subject, will be in a position to do for the dental profession a service that will be worth a great deal to us, and that is, in all of my history as a practitioner of dentistry, while I have had intimate personal relations with many physicians, and physicians of all kinds, men who are specialists in all diseases, but particularly in this disease, I never have had a physician write me a note or speak to me personally in regard to a patient that he knew I was treating, and inform me of the condition of such a patient. How many men in this room have had a physician do that for them? Seven or eight hands were raised, I am glad to know that there are a good many of you that have; it has not been my experience. It is considered by the physician that conditions of this sort are between the physican and his patient sacred, but that is no sufficient reason why, when it is known that in the dental chair there are so great opportunities for the transmission of this disease—that there should be no discourtesy on the part of the medical profession in conveying such knowledge, in confidence, to a dentist whom he knows is treating the patient that is afflicted in any stage, with this disease; particularly, if it is during the time when the disease is liable to be transferred by dental operation, or where the dentist himself would be liable to become infected with that disease. If physicians will take us into their confidence, as it seems to me they should, because of the fact that from the very nature of things we cannot become expertly intelligent on this subject, so that we can make these diagnoses on the instant when these patients present to us, it seems to me that the medical profession will do us an everlasting favor, and they will in that way also do something to prevent the spread of this disease, if it is being spread, as many of the articles that I have read from time to time have stated, by dental manipulation or by the use of dental instruments. I myself do not believe that the man who practices dentistry, with modern knowledge of antiseptic cleanliness, would spread disease of any sort through his instruments, if he is reasonably careful. If he is reasonably careful so far as ordinary hygienic and antiseptic precautions are concerned, he will spread no disease through the use of infected instruments, but it is possible, however careful we may be, that some disease may be transmitted in this way, and we may be responsible for dreadful results when really the innocent and unintentional instrumentality for doing so. It seems to me that the medical profession owes us something in this respect. Medical practitioners are in a position to know of these conditions, and when they know that patients of theirs are suffering in this way and they are at the same time being treated by dentists or oculists, or any one else, where they would be likely to contaminate our instruments, or be the means of spreading the disease to some other, it seems to me that we ought to be considered in this connection, and I hope that Dr. Varney, when he is speaking of this subject to his medical confreres will not forget that point. Every man, optician, oculist or any other man who is doing anything outside of the regular medical work, and who is supposed to be an expert in these lines, ought to be considered in connection with this matter.

Now as to the means which the dentist may adopt. I was in hopes that Dr. Oakman would take up this part of the subject and tell us how we may avoid being infected ourselves in handling patients. I know that he must have had more experience than most of us in his surgical work. How is it possible for the dentist to sterilize not only his hands in preparation for operations upon people generally, and particularly upon suspicious cases, and how is it possible for us to sterilize our instruments and to avoid any possible inoculation of our patients in this way?

I am in the habit of sterilizing my instruments after every operation, of every kind, and most of us do that thing carefully and as religiously as we know how. I depend largely upon 5 per cent. solutions of lysol as my sterilizing agent preliminary to a boiling water bath as a final cleansing and sterilizing agent. These two agents it seems to me are sufficient. Where I have cases that I am in doubt as to the efficacy of this method of sterilization, and my instruments are capable of being heated, of course I employ dry heat direct; but if we do this with all of our instruments, some of them would be ruined. In extreme cases I have resorted to the use of Formaldehyde and Alcohol, a 25 per cent. solution of Formaldehyde in Alcohol, with a little borax to prevent rusting.

The method of sterilizing the hands that has been presented to us by Dr. Varney with the Calomel Paste is perhaps all right for emergency cases, or for cases where we know we are liable to inoculate and are dealing with an extreme condition; but for the ordinary sterilization of the hands it would be too severe on our hands. I do not believe we could use such an ointment as that because it would destroy the tactile sense in our fingers, our nails would become all broken up and hard and crack, so that our hands would not be sightly to put in to anybodys' mouth; it would be out of the question for us to use agents of that kind it seems to me.

I have sometimes tried to sterilize my hands with a bichloride of mercury solution, but it is very hard on my hands, and I know in general it is not an agent that you can make use of, because we must have our hands in a presentable condition when we are handling our patients. We must not destroy the delicate, sensitive touch, which really is essential to the handling of our instruments in a definite and delicate manner, so that any agent that would do this and would injure our tactile sense we cannot use at all. Of course different persons can use different things. The cuticle of some hands will stand sterilization that others will not, but I find that the liquid soap used with hot water is about as good a sterilizing medium as I can find. It keeps my hands in good condition and I feel confident that it is effective and sufficient for my purposes. Sometimes I use a solution that Dr. Spalding gave me, or recommended, a solution of Phenol in Rose Water and Glycerine—I do not remember just the formula of it now. It is an excellent thing to use, not only to sterilize your hands but it keeps them in good condition, keeps them soft and pliable, and I feel with the use of this soap and an agent of this sort I am protecting my patient and myself as well as I can under the circumstances. I wish Dr. Varney would tell us whether it will be sufficient in these extreme cases, where there is definite infection, and where the virus from this infection is so virulent that even if it is contained none of the spirillum, would be sufficient to infect. But this Calomel Ointment, I am afraid, would not be an agent that we could use for any great length of time; it may be for an occasional and severe case the one that we would be glad to use.

Dr. C. H. Oakman.—Dr. Hoff suggests that Calomel might not be tolerated by the hands, I don't really know how it will act. I am going to try it, combined with lanoline, which is not as presentable as some things, and I would think that cocoa butter might be used instead. I do not think any preparation for sterilization of the hands would be just the thing when it contains glycerine, for we know that glycerine will extract the moisture from the skin and consequently dry up the hands. Personally I can use bichloride of mercury, 1-3,000, year in and year out; for the last fifteen years I have used bichloride 10 or 30 times a day.

How many dentists religiously disinfect their hands before or after every operation? I do not think soap is sufficient, because about the strongest antiseptic you can get in the soap is peroxide. You may have other ingredients, but I doubt, from my experience at least, if you can use the stronger antiseptics combined in the soaps. I possibly could stand, as I said, bichloride; I do not know how it would be with most of you. I know dentists for years past that have never used an antiseptic on their hands nothing more than soap. Now that does not seem to be a fair deal for our patients, as we are working in syphilitic mouths all the time. I doubt if any of us spend a day without running across some syphilitic cases; I know there is not in my office a day that I do not see three or four. It may be that cases of syphilis are sent to me more frequently for treatment of the gums. We know that patients affected with syphilis will almost invariably have a pyorrhoeal tissue; that in my experience is almost always the case, and I think that physicians should not send these patients to us in the primary stage. Many dentists say, I would not work for patients in any stage. If we do not, we are not doing our duty in the healing art. I am willing every day to see as many syphilitic patients as I can, because the relief you give them and the healthy condition you put their mouth in, and the gratification you get out of it is well worth the while. I think that Dr. Hoff struck a keynote when he said the physician should have confidence in the dentist. There should be some way that he may communicate this information to us without a breach of professional etiquette. We are then forewarned and are more careful, and are more likely to use instruments set apart for that purpose and used for no other purpose. I think the physicians are learning, at least the better men, to treat the dentists with more consideration. I will just relate an incident to show what the dentist might be up against.

A patient that I was treating for an ordinary lesion appeared in three days with a chancre on the upper left lip, about the size of a white bean, and she came again in a few days and it had that hard chancre appearance. I was a little suspicious and advised her to go to her family physician. This was some ten years ago. (I would have sent her to a specialist should it occur now, if I had anything to do with the sending.) What I got for my pains was a terrible roasting. I asked, "By the way, who was your family physician?" "Dr. so and so." "I would go right to Dr. ——— and ask him what he thought of this, and he may call me up and talk with me." The first thing the girl said was, "I have been to the dentist, and I think he stuck an instrument in to my lip." Remember it was only three days, and I knew I had not punctured her lip with an instrument. He said, "That is the way dentists do; I have a lot of patients come with things like that on their lips." She became frightened, called up her beau, and he came to see me, very much excited, and wanted to know how this occurred. I put on my hat and coat and went and hunted up Mr. Physician; before I went I 'phoned, to be sure he would be in but he would not see me. The girl came in about six weeks later and had some of the secondary symptoms, from what she said, and kept on with the treatment. Later on her finger nails became affected. I knew that I was blamed for the condition, and said to her, "Why don't you go to a good doctor?" "Isn't he a good doctor?" "I don't know for sure, but you had better go to a man who is well versed in this work." He lost the patient, and later I was vindicated; both she and her beau agreed that I could not have done it, but it goes to show what an unpleasant position you might be placed in, so when you have these suspicious looking sores, don't put off the time of sending them to some good physician or specialist for treatment.

DR. J. M. THOMPSON.—I would like to ask Dr. Varney if he thinks that all cases of pyorrhoea alveolaris are an evidence of some syphilitic taint somewhere. Dr. Curtis, of New York, claims that all inflammation of this class are due to syphilis.

MR. CLARK.—From some of the questions asked while I was demonstrating the spirochaeta pallida with the microscope it seems that the distinction made between the organism as demonstrated and the antigen used in making the serum test is not clear and is here rather confusing.

This serum test deals with the products of the organism in the system, and not with the organism directly. Dr. Varney spoke of the antigen, but did not explain what the antigen really is.

An antigen may be a suspension of an organism or it may be the toxins or the poisonous products of that organism. In this case we use the extract of a syphilitic liver which extract does not contain the spirochaeta; it simply contains the products of the spirochaeta, and here we are dealing with the products of the organism and the products or bodies in the serum produced by the system to counteract the effects of the invading organism.

DR. LAUPPE.—I would like to ask, how long is it necessary to leave the mercuric paste on the hands, three hours, did I understand you to say, or one hour?

DR. VARNEY.—One to three hours after inoculation, but if the paste was on before any inoculation, in any suspected case, there would not be any infection likely to take place.

I am sure I am very much gratified by the liberal way in which you have taken up this subject. I realized that it was vast and a good bit of it was new, but yet I felt that, as Dr. Hoff has stated, if I could give you something that was embodied in the recent research work of this disease that you would be able to apply it clinically, and in your discussion you have proved this.

In regard to the virus, or statement in regard to the virus as producing the disease, when the organism was not able to pass through the filter, that is an experiment of Janckos and he really does not explain how that comes about, but it is possible for the toxic to produce the disease. He produced the disease in a number of animals from the filtered virus that passed through the Chamberlin filter.

We could ask many questions, because this field is one recently opened up, and there is a vast amount of it, as you can readily see, that is yet unexplored, and a vast amount of research must be further gone over before many of the questions that arise can be answered.

Dr. Oakman referred to the Taurin reaction, or precipitation reaction, which I was especially interested in and which attracted my attention a year ago in the examination of a number of bloods, not only of positive syphilitic patients but doubtful syphilitic cases, both in private and clinic practice in one of your larger institutions of the state. As I stated in my paper, the blood examinations with this test were carefully controlled and all bloods were examined by number, with no knowledge of the clinical history. A large percentage of them were examined in that way, and the result of this precipitation reaction, after it was read, was confirmed by the history of the case. In this way the worker eliminates the danger of being misled or going off in a wrong direction, which might occur to the enthusiast. I was attracted by the character of the precipitation, flocculent precipitation taking place in shorter time than normal, in normal sera and larger quantity, and I feel, with experience thus far, that the Taurin in precipitating this globulin in a syphilitic serum is of some value and is of great assistance to me daily in connecting one more link in the chain of evidence in arriving at a diagnosis. I would not make a diagnosis on the precipitation test alone in a doubtful case. I would not make a diagnosis on one positive Wassermann reaction in a doubtful case; I would run two or more at different intervals of time, we will suppose, from the first reading, if the case was one of doubt as to infection, with no history.

Dr. C. H. Oakman.—What percentage of cases of tabes can you detect?

Dr. Varney.—Between 50 and 60 per cent. of Tabes dorsalis show positive reactions with the Wassermann test; but they are considered now by internists to be 100 per cent. syphilitic in their etiology.

Dr. Wakman touched upon a very important field which I did not have time to take up, and that is, the treatment of the gums in the syphilitic. It is a routine practice of mine, before starting the patient upon any treatment, to examine thoroughly the condition of the gums, to detect, if possible, any recession or pyorrhoeal condition. If such a condition exists the man who starts treatment before these gums are cared for will be handicapped in controlling the disease. It is not mercury that causes the teeth to loosen or to fall out. One of the most troublesome traditions that I have to overcome is: "Mercury" gets into my bones, my teeth get loose," they say. It is not mercury; it is the condition of the mouth before the mercury was given, many of my patients are being sent to dentists for the treatment of the gums, before any mercury is administered and then carefully instructed regarding the importance of keeping the buccal cavity in a healthy condition during mercurialization.

Dr. Lauppe.—In case the gums are healthy and the treatment is that of mercury, will the teeth get loose and gums become inflamed from salivation.

Dr. Varney.—No, not unless the case becomes salivated and that is only transitory. Treatment may be vigorous if the mouth is in a healthy condition.

Dr. Hoff, I think, has brought up one of the most important phases of interest to us all, that of the physician notifying the dentist of the nature of the infection in that mouth. The patient may not have an evidence of any syphlitic manifestation in the buccal cavity; one may not mistrust a patient in the least; He may come from one of your best families, perhaps a pillar of a church; in fact, we know that this disease is no respecter of person, it is in all walks of life. If I have impressed upon you the importance of better caring for yourself, to prevent any possible chance of infection, and then of transmitting it to patients, which I think is far less risky than your own possible inoculation, I will feel that something is accomplished.

I mentioned in my paper the scientific fact that the organism does not need to enter the blood stream; all that is necessary is that it enter a moist weeping fissure of the skin; in that case, with the least bit of serum one is liable to have an initial lesion. If you were in a position to see the number of cases that have been innocently infected in our city you would realize how keenly I feel the importance of subjecting every mouth that has the slightest break in the mucous membrane, if it is only a crack in the corner of the mouth, to further examination and suspicion. Look out for the slightly reddened, smooth placks on the tongue where the epithelium is gone; look out for those little gray patches in the mouth that the patient has not noted because they are not sensitive, and if the patient's attention is called to it, they have "canker sores," that patient should be under suspicion without conveying any knowledge about the diseased condition. The fore finger of the right hand is a most likely focus for infection, and your profession are most often infected here by not careful sterilization of the hands before and after operation, from patients who have no evidence of the disease.

In regard to calomel paste, I mentioned that because I thought I ought to put something in my paper that could be applied practically and that has been proven, scientifically, by a number of men, Metchnikoff and Neisser and others, that a preparation of one part calomel to three parts of lanoline, will abort the disease, even after inoculation; it will destroy the organism. If you have suspected cases and rough epithelium about your finger nail, calomel paste should be applied, I would not care what it tasted like, to that patient. I think the lanoline is very protective to the epithelial layer and does not cause hardening of it. It was mentioned only to be applied, of course, when you have an area on your finger that might possibly allow the entrance of the infection, and when you have a suspicious mouth to work in.

I mentioned the fact that the organism lives only a short time out of the body, and that accounts for the fact that no more of us are infected daily. The organism dies very rapidly, and is not transmitted from common articles that we use as often as we might suppose. Lysol I think, has sufficient disinfecting features to destroy the organism directly after any operation. Unless mouth fluids are allowed to dry or enter the cracks of your hands and remain there for some time, most solutions will take care of them. I really think that potash or soda soaps, that ether soaps, really answer most purposes in disinfecting, unless the patient's mouth is suspicious. Another soap not mentioned to-night is iodide of mercury soap, although rather harsh on the epithelial layers if used often.

Dr. Thompson asked the question as to whether pyorrhoea is an evidence of some syphilitic taint. It is difficult to tell how many of us can go back three generations in our own family history, without some taint of syphilis. During the Revolutionary times the disease was most prevalent in Europe, and it was laid to the American's doors that the British soldiers brought it home, and you know that in France it was a national question at one time, so prevalent was the disease. Kings and queens and the royal family have syphilis, so that, as I say, it is not possible to go back many generations without getting some taint of syphilitic manifestations, perhaps, in our own make up, so that these diseased conditions of the gums may be, a good many of them, due to syphilitic infection, and we cannot get much absolutely accurate evidence to prove such a statement.

## Figures and Tables

**Figure f1:**